# Investigating the drivers of the spatio-temporal patterns of genetic differences between *Plasmodium falciparum* malaria infections in Kilifi County, Kenya

**DOI:** 10.1038/s41598-019-54348-y

**Published:** 2019-12-13

**Authors:** Josephine Malinga, Polycarp Mogeni, Irene Omedo, Kirk Rockett, Christina Hubbart, Anne Jeffreys, Thomas N. Williams, Dominic Kwiatkowski, Philip Bejon, Amanda Ross

**Affiliations:** 1 Swiss Tropical and Public Health Institute, Basel, Switzerland; 2 University of Basel, Basel, Switzerland; 3 Kenya Medical Research Institute-Wellcome Trust Research Programme, Kilifi, Kenya; 4 Wellcome Trust Centre for Human Genetics, University of Oxford, Oxford, UK; 5 Department of Medicine, South Kensington Campus, Imperial College London, London, UK; 6 Wellcome Trust Sanger Institute, Wellcome Genome Campus, Hinxton, Cambridge UK; 7 Centre for Tropical Medicine & Global Health, Nuffield Department of Clinical Medicine, University of Oxford, Oxford, UK

**Keywords:** Computational science, Epidemiology, Statistics, Genetics research

## Abstract

Knowledge of how malaria infections spread locally is important both for the design of targeted interventions aiming to interrupt malaria transmission and the design of trials to assess the interventions. A previous analysis of 1602 genotyped *Plasmodium falciparum* parasites in Kilifi, Kenya collected over 12 years found an interaction between time and geographic distance: the mean number of single nucleotide polymorphism (SNP) differences was lower for pairs of infections which were both a shorter time interval and shorter geographic distance apart. We determine whether the empiric pattern could be reproduced by a simple model, and what mean geographic distances between parent and offspring infections and hypotheses about genotype-specific immunity or a limit on the number of infections would be consistent with the data. We developed an individual-based stochastic simulation model of households, people and infections. We parameterized the model for the total number of infections, and population and household density observed in Kilifi. The acquisition of new infections, mutation, recombination, geographic location and clearance were included. We fit the model to the observed numbers of SNP differences between pairs of parasite genotypes. The patterns observed in the empiric data could be reproduced. Although we cannot rule out genotype-specific immunity or a limit on the number of infections per individual, they are not necessary to account for the observed patterns. The mean geographic distance between parent and offspring malaria infections for the base model was 0.4 km (95% CI 0.24, 1.20), for a distribution with 58% of distances shorter than the mean. Very short mean distances did not fit well, but mixtures of distributions were also consistent with the data. For a pathogen which undergoes meiosis in a setting with moderate transmission and a low coverage of infections, analytic methods are limited but an individual-based model can be used with genotyping data to estimate parameter values and investigate hypotheses about underlying processes.

## Introduction

Ultimately the spatial and temporal patterns of malaria infections at the community level are made up of movements of individual mosquitoes, human hosts and the parasites inside them. Understanding how malaria infections spread locally and the processes leading to the observed spatial and temporal distribution patterns is important for the design of interventions aiming to reduce and interrupt transmission by targeting foci, to estimate the spread of drug resistance, or to prevent resurgence^1,2^.

Investigating genetic patterns can give insights into how infections spread^3–7^. The availability of high-throughput genotyping methods has enabled the genetic characterization of the malaria parasite, identifying polymorphisms on the genome that can be used to infer the degree of genetic relatedness between infections^5,6^. Using single nucleotide polymorphism (SNP) genotyping, some studies show clustering of parasites into distinct sub-populations between Thai and African^8–10^, West and East African^9,11^, and Asia and African isolates^9,11^ while others report persistent clustering of genetically identical parasites across years in Senegal^8^. On a sub-national scale, a study from school surveys in western Kenya, an area of high malaria transmission, found no spatial structure to genetic relatedness over geographic distance in a well mixing parasite population^4^. Studies have also used genetic information to estimate the risk of imported infections^7,12^, to estimate transmission networks in Swaziland^13^, to provide confirmatory signals of the decline of transmission in Senegal^14,15^, and to describe the relatedness of parasite pairs between proximal clinics at the Thai-Myanmar border^16^ and focal transmission in Zambia^17^. However, there are few studies focusing on parasite genotype patterns in the community on a fine scale, such as within a district.

A recent study investigated *Plasmodium falciparum* genotypes in two districts in Kenya, and in one district in The Gambia^3^. All possible pairs of observed parasite genotypes within each site were formed and the number of SNPs different calculated. In the sites with longitudinal data, the effect of the time interval on the pairwise SNP differences was modified by geographic distance. For short distances, the number of SNPs different increased with time, but saturated as distance between parasites increased^3^.

While these studies provide information on the distribution of malaria parasite genotypes over space and time with qualitative inferences on parasite mixing, the underlying parameters and processes leading to these patterns have not been well established and the geographic distances between parent and offspring infections remain unclear.

In many malaria endemic areas, vector movement is likely to be the most frequent mechanism for the spread of infections. Studies have shown that mosquitoes tend to fly relatively short distances from their breeding sites in search of blood meals^18–20^, depending on the distance to human settlements, the availability of easily accessible hosts, and whether any barriers to movement exist^19,20^. In Kilifi, mosquitoes were recaptured in households within 0.7 km of larval habitats up to 14 days after release^18^, while in other settings flight distance from release points including breeding sites was reported to range from less than 100 m to 3 km^21–24^, or further^25^. However, there is limited information on the distances between households moved by the mosquito from the initial bite when the mosquito becomes infected to the subsequent bites when further humans are infected. After a blood meal, the mosquito looks for a resting place to digest the blood and develop eggs and after two to three days, searches for a breeding site to oviposit before seeking another host. The extrinsic incubation period (EIP) takes approximately 10 to 12 days for the mosquito to become infectious, corresponding to roughly two or three feeding cycles. Human movement may also inform malaria distribution patterns^26,27^, especially in populations with low transmission and high rates of imported infections^27,28^. Hence, simply knowing the dispersal distance from breeding sites does not lead to a calculation from which we can predict the mixing of parasite genotypes.

The interaction between time and geographic distance on genetic differences found by Omedo *et al*. raises questions about the processes behind it. The explanation may be stochastic drift or may require additional processes such as genotype-specific immunity. The effective repertoire of antigenic variation may be reduced by immunity raised from the previous infections with the same or similar genotype. Deliberate infections of the same strain in naïve adults as malaria therapy led to shorter patent infections with lower parasite densities than infections with a different strain^29,30^. It is not also known if there is a limit on the number of infections that one person can have at a time. There is saturation in the mean multiplicity of infection (MOI) detected with transmission intensity^31,32^ but this may be due to the limits of detection. This would decrease the chance of large numbers of similar infections circulating in the same household. Recombination may occur in the mosquito during meiosis if gametocytes from multiple infections are drawn up in a blood meal. Recombination is likely to be dependent on the intensity of transmission and the relative gametocyte densities of the infections^33,34^ and may play a role in the distribution of parasite genotypes across time and space, mainly through reshuffling of alleles that are already present.

Our aim is to assess which processes and parameter values are consistent with the observed spatial and temporal patterns of parasite genetic differences observed in Kilifi County, Kenya^3^. We develop a stochastic simulation model and fit it to the observed number of SNPs different between pairs of parasite genotypes. We estimate the distance between parent and offspring malaria infections, and investigate the role of recombination, imported infections, and hypotheses about functions of immunity. We evaluate the ability of this method to recover known parameter values using simulated data.

## Methods

### Data sources

#### Study site

The study was conducted within the area covered by the Kilifi Health and Demographic Surveillance System (KHDSS) on the Kenyan Coast. The area covers 891 km^2^ with a population of approximately 260,000 people^35^. Most of the residents live off small scale farming and fishing. Malaria transmission in Kilifi is seasonal^36^, with long rains between April and June and short rains between October and December. The malaria prevalence reported from both health facilities and community surveys declined over the period of the study^37,38^, attributed mainly to increased coverage of control interventions, improved case management and environmental changes^37,39^.

#### Sample collection and genotyping

The details of the sample collection and genotyping process have been described elsewhere^3^. Briefly, 1602 *P. falciparum* -infected blood samples were collected over 14 years between 1998 and 2011. Of these, 1259 (79%) samples were from hospital cases in children between three months and 13 years old, 195 were from community surveys with participants between 3 weeks and 85 years old and 148 were from short term laboratory cultured samples^3^. The residence location of the participant providing the sample was recorded. The samples were genotyped using the Sequenom MassARRAY iPLEX platform^40^. Up to 256 SNPs were typed in each sample, across the 14 parasite chromosomes^3^. Sample success rates and genotyping pass rates were determined for each sample and for each SNP. SNPs and samples with high failure rates (>30%) were excluded from the final analyses^3^.

#### Data preparation

For the present study, the number of SNPs included, *n*_*s*_, is 53. We selected bi-allelic SNPs with a minor allele frequency (MAF) of greater than 5%. We denote the presence of a major allele at each SNP position by 1 and the minor allele by 0. Each infection is then characterized by a string of zeros and ones to represent the observed alleles at each of the selected loci. The base-10 number is generated from this base-2 number to give a unique identifier for each infection genotype. We assume that the genotypes represent single infections.

#### Calculating pairwise time, distance and SNP differences

Following Omedo *et al*.^3^, the observed parasite genotypes were paired and the number of SNPs different between them used as the measure of genetic difference. Pairwise differences in time in days and distance in kilometres were also computed.

### Modelling strategy

We aimed to determine which processes and parameter values were consistent with the observed patterns of genetic differences over time and distance in Kilifi, where the observed numbers of SNPs different were fewer for parasite pairs with both a shorter time interval and a shorter geographic distance apart^3^.

We developed a simple individual-based stochastic simulation model describing how malaria infections are transmitted over time and space. The acquisition and clearance of individual blood-stage infections in people living within homesteads was simulated. The site-specific inputs such as house density, number of people per house, the total number of infections and prevalence over time were derived from published estimates from Kilifi^35,37^. We extended the base model to a set of model variants including the effects of imported infections and hypotheses about whether there needs to be genotype-specific acquired immunity or a limit on the number of current infections per individual for model predictions to be consistent with the patterns in the data.

We objectively measured how well the model variants and parameter values fit the data using a likelihood function which compares the numbers of SNPs different in pairs of blood samples to those from pairs of simulated infections drawn from the same time points and closest locations. We estimated the mean distance between parent and offspring infections and the probability of recombination in multiply infected individuals.

### Base model

We refer to the most simple simulation model as the “base model”. The simulation model was seeded with a number of initial current infections. Households and people within households were randomly selected for each initial infection. The initial infection genotypes were generated by randomly selecting alleles based on the MAF in the observed data at each locus for the selected SNPs.

At each five-day time-step, each current infection could give rise to new infections. The number of new infections for an infection *i* of age *a* at time *t*, *n*_*iat*_, was stochastic and was drawn from a Poisson distribution with mean *μ*_*at*_, so that *n*_*iat*_ ~ *Poisson*(*μ*_*at*_). The mean number of new infections, *μ*_*at*_, was the product of the relative infectiousness to mosquitoes of an infection of age *a*, *I*_*a*_, and a constant, *m*_*t*_, so that *μ*_*at*_ = *I*_*a*_*m*_*t*_ (Table 1). Values of *m*_*t*_ were fixed to generate the number of infections which was set to the estimated prevalence in Kilifi multiplied by the number of individuals and the MOI corresponding to the prevalence and estimated by a systematic review (^32^, Malinga *et al*. submitted). *I*_*a*_ followed a decay function which was derived from the estimated period of higher infectiousness of an infection^41^.

**Table 1:** Quantities in the simulation model.

Quantity	Description	Units of measurement
*n*_*s*_	number of SNPs	positive integer
*a*	age of an infection	five-day time-step
*t*	time from start of simulation	five-day time-step
*n*_*iat*_	number of new infections for infection *i*of age *a* at time-step *t*	Integer, greater or equal to zero
*μ*_*at*_	mean number of new infections for an infection of age *a* at time-step *t*	Numeric, greater or equal to zero
*m*_*t*_	mean number of new infections for an infection at time-step *t*	Numeric, greater or equal to zero
*I*_*a*_	relative infectiousness to mosquitoes of an infection of age *a*	Numeric, 0 ≤ *I*_*a*_ ≤ 1
*p*_*m*_	probability of mutation for one allele per infection cycle	Probability
*p*_*c*_	probability of clearance per five-day time-step	Probability
*p*_*r*_	probability of recombination conditional on multiple infections in a host	Probability
*σ*	parameter for distance between parent and offspring infection, (mean = $\sigma \sqrt{(2/\pi )}$	Kilometres

Each new infection was assigned a genotype. The genotype was the same as that of the parent infection except for two processes, mutation and recombination. Mutations may occur in the new infection, where each allele mutated with probability *p*_*m*_. In individuals with more than one current infection, recombination occurred with probability *p*_*r*_. If a new infection was the product of recombination, then the infection with which it recombines was drawn at random from the remaining infections in the same individual. The two infections may have the same genotype, in which case selfing occurs. Recombination hotspots for different chromosomes have been reported^42,43^, but we assumed that the probability of a break is equal along the length of the chromosome and each chromosome could break in only one place^42^. The new recombinant infection had one genotype in the base model due to computational demands. A model version allowing four different sibling genotypes as a consequence of meiosis was also included. However, this version substantially increased complexity for little gain in accuracy: infections are expected to lead to zero or one subsequent infection due to declining transmission, the chance of selecting a pair of simulated infections arising from the same recombinant infection is small and the number of infections is tailored to that of Kilifi thus either co-infections or separate infections would allow a similar number of recombinations. The effect of a model not capturing the full complexity of the recombinations in the observed data would likely be conservative by increasing the imprecision of the estimates. We assumed a constant probability of clearance of each infection in a five-day time-step, *p*_*c*_.

**Figure 1:**
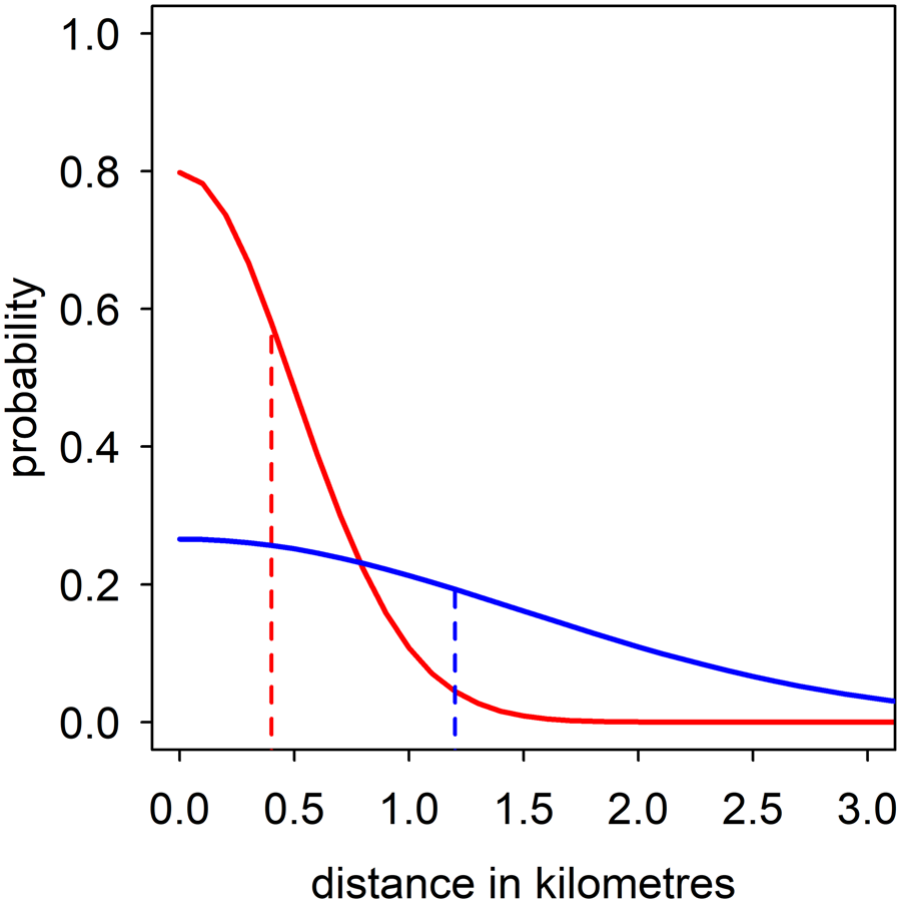
Examples of the half-normal distribution probability density function for positive values of the distance between parent and offspring infections. The dotted lines mark the mean of the distribution; Red line = 0.4 km, Blue line = 1.2 km.

Each new infection was also assigned a homestead. The homestead was selected randomly, with probability according to a function of the distance from the location of the current infection. We assumed that the probability follows a normal kernel, simulating diffusion. The cumulative distance of Brownian motion at a specified time is given by the kernel for positive distances of a normal distribution with mean of zero and a standard deviation denoted by *σ*. The mean distance between parent and offspring infections is given by $\sigma \sqrt{(2/\pi )}$, and 58% of distances are shorter than this value (Fig. 1). The probabilities are not scaled so that an isolated household has a lower chance of transmitting to other households than does a house which is close to other houses. An individual within each homestead was selected at random to host the new infection.

### Model variants

We included alternative model variants to investigate the effect of imported infections and whether hypotheses about specific immunity are supported by the observed data. We simulated (i) the base model, (ii) the base model with imported infections, whose alleles were chosen with probability equal to the individual allele frequencies, (iii) the base model with a limit on the number of current infections: new infections cannot establish in a person if they already have the maximum permitted number of infections, (iv) the base model with acquired immunity to recently seen genotypes: a new infection cannot establish in a person if they have already seen a number of genotypes with a limited number of SNPs different in a recent time period, (v) the base model with heterogeneity in transmission at the household level, and (vi) the base model with each combination of pairs of (ii)–(v).

We did not simulate acquired immunity in individuals in general, but only specific acquired immunity to recently seen genotypes since it may affect the spatial and temporal distribution of parasite genotypes.

### Model inputs

#### The area simulated

Population and household density estimates were derived from the KHDSS surveillance reports (Table 2). We required 20 homesteads per kilometer squared, with eight residents per homestead. Using the estimated parasite prevalence in Kilifi^37^, and an estimate for MOI of 1.5 corresponding to the prevalence^31^, we would require approximately 200 malaria infections per square kilometer at the beginning of the study period declining to 25 by the end. For the whole study area of 891 km^2^, however, this would have high computational demands. Instead we simulated an area of 9 km by 9 km. This is the largest size that could be run within 24 hours for the base model and comfortably covers the range of zero to 3 km where the interaction between time and distance was observed^3^. We included 1800 homesteads each with eight people. The number of simulated infections present in the 9 km by 9 km square at the same time is around 13,000 at the beginning of the study period declining to 2,000 at the end of the study.

**Table 2:** Inputs to the model.

Parameter	Value	Reference
***Kilifi Characteristics***		
population density	260,000 people	^35^
area size (in km^2^)	891	^35^
median number of people per homestead (IQR)	8 (6–11)	
median homestead density per km^2^ (IQR)	20 (7–58)	(a)
estimated parasite prevalence	60% in 1998; 10% in 2011	^37^
number of infections per km^2^	200 in 1998, 25 in 2011	(b)
mean number of new infections per five-day time-step(*μ*_*t*_)	Varies	(c)
***General fixed inputs***		
probability of clearance per five-day time-step, *p*_*c*_	0.025	(d)^44,60^
probability of mutation per SNP, *p*_*m*_	2.0 × 10^−7^	(e)
relative infectiousness of an infection by age of infection, *I*_*a*_	Varies	(f)^41^

(a) The co-ordinates for households in the simulated 9 km by 9 km grid were randomly drawn from two independent uniform distributions.

(b) The expected number of infections was derived from microscopy and RDT prevalence data and multiplied by the MOI corresponding to the prevalence in a systematic review (^32^, Malinga *et al. submitted*).

(c) The values of *μ*_*t*_ are fixed according one of the phases of the simulation to produce the correct numbers of infections estimated for Kilifi in (b).

(d) Assuming an exponential decay function, a probability of clearance of 0.025 per five-day time-step corresponds to a mean duration of infection of 200 days^56–58^.

(e) The probability of a mutation per base pair per generation has been estimated to be 1.7 × 10^−9^ ^59^. We multiplied this by the estimated number of generations in the liver stage, for the gametocytes and in the asexual blood-stage, each of 48 hours, before the mature gametocytes are taken up in a blood meal. The probability of at least one mutation per SNP per infection cycle is then approximately 2.0 × 10^−7^.

(f) Assumes an exponential decay function estimated from a period of higher infectiousness of an infection (probability of infection >5%)^41^.

At the edge of the grid, infections have a restriction on the direction in which they can move. To avoid artificially pushing too many infections back towards the centre of the grid, we specified that current infections in a 5% border at the edge of the 9 km by 9 km grid give rise to half as many new infections per time-step. We used the central 6 km by 6 km area for fitting, allowing a 3 km border to minimize edge effects on the pattern of genetic differences.

#### Number of infections and model run time

The model was initialized with 13,000 current infections, distributed randomly across homesteads and to individuals within the homesteads.

The model running period was divided into three phases. To achieve the required numbers of infections over time, values of *m*_*t*_, the multiplier for the number of new infections for a current infection in time-step *t*, were set to be constant within but to vary between the different phases of the simulation. The value of *m*_*t*_ also needed to vary slightly according to the input parameter values, in order to reach the required numbers of infections in the study area. The initial phase was made up of 1000 five-day time-steps with *m*_*t*_ set to lead to an increasing number of current infections. This phase was not necessary if the number of initial infections was equal to the number required at the start of the study period. The second phase had 1000 time-steps with *m*_*t*_ set to keep the number of infections constant as a warm-up period to allow the structure in the genetic differences to develop, and the study period phase covered the 850 five-day time-steps for the observation period with *m*_*t*_ set to reproduce the decline in the number of infections observed in Kilifi.

#### Sensitivity analysis

We conducted sensitivity analyses to investigate the influence of input parameter values where the real value was unclear. These include varying the amount of clustering of the distribution of households within the study area, the number of initial current infections, the warm-up period and the size of the simulation area.

There is also uncertainty surrounding the genotyping of infections from samples collected from individuals with multiple *P. falciparum* infections. It may be that a dominant infection is captured during genotyping, or that the process selects different alleles from different infections. For the base model, we assume that only one of the infection genotypes is sampled from individuals with multiple or recombinant infections. However, we investigate the effect of detecting a mix of alleles for different SNPs from different infections in the same person.

### Fitting the models to the data

The model was fitted to the data by comparing the number of SNPs different between pairs of simulated infections to the number of SNPs different between the observed infections. The simulated infections were matched to the observed infections on time-step and location.

The study area was divided into 6 km by 6 km grid squares. The locations of each observed infection in each square were transformed from GPS co-ordinates into the co-ordinates of the 6 km by 6 km area, and the date that the sample was collected converted into the time-step of the simulation. We nest the 6 km by 6 km square within the 9 km by 9 km simulated square. The 3 km buffer serves to provide the correct spatial structure for infections within the nested area.

For each observed infection, a number of simulated infections were selected at exactly the same time-step. Infected individuals closest to the location of the observed infection were identified, and a maximum of one infection per individual was sampled. To balance the effects of stochasticity, the requirement for selected infections to lie close to the observed location and computational demands, five simulated infections were selected for each observed infection per simulation, and each simulation was repeated with ten random seeds.

Within each 6 km by 6 km square *k*, we formed all possible pairs of observed infections, and for each pair *j*, calculated the observed number of SNPs different, *d*_*jk*_. We then calculated the number of SNPs different in the simulated data, *q*_*jrk*_ within each 6 km by 6 km square *k*, for each pair *j*, and for each of the 10 replicates of the simulated infections *r*. We gained the mean proportion of SNP differences for the replicates $\,{\bar{q}}_{jk}/{n}_{s}$. The Binomial log likelihood was used to quantify the amount of support from the data, calculated by summing over all the pairs *j* within each square *k* and over all the squares.

$$ll=\sum _{k}\,\sum _{j}\,{d}_{jk}\,\log (\frac{{\bar{q}}_{jk}}{{n}_{s}})+({n}_{s}-{d}_{jk})\log \{1-(\frac{{\bar{q}}_{jk}}{{n}_{s}})\}$$

The observed pairs are not independent given that one observed parasite genotype contributes to multiple pairs of data. Therefore, 95% confidence intervals were obtained by using bootstrap resampling of the observed number of SNP differences.

### Method evaluation

We evaluated how precisely the method could estimate parameter values. We simulated data with known values for *σ*, the parameter for the distance between parent and offspring infections, and probability of recombination, *p*_*r*_, and then applied the method to the simulated data to see how well the known values could be recovered. We used the reference scenario with the base model (Table 3), but with a constant rather than declining number of infections over the study period.

**Table 3:** Simulated scenarios.

Parameters to be estimated by a grid search	Value
parameter for the distance between parent and offspring infections (in kilometers)**	0.10, 0.20, 0.30, 0.40, 0.50, 0.60, 0.70, 0.80, 1.00, 1.20, 1.50, 2.00, 2.50, 3.00
probability of recombination resulting from multiply infected individuals	0.01, 0.50, 1.00
**Parameters included in the model variants**	Value*
imported infections per 1000 people per year (model variant (ii))	**0**, 5, 10
total number of current infections per person (model variant (iii))	**no limit**, 20
maximum number of recently seen similar genotypes for new infection to fail to establish (model variant (iv))	**no limit**, 10
number of SNPs different for defining ‘similar’ genotypes (model variant (iv))	**0**, 10
number of time-steps for counting recently seen similar genotypes (model variant (iv))	**0**, 40
heterogeneity between houses in transmission (model variant (v))	**No heterogeneity**, log normal distribution with mean *μ*_*at*_ and standard deviation of 0.01

*The base model scenario is indicated by bold font. **The mean of a half-normal distribution is given by $\sigma \sqrt{(2/\pi )}$.

### Scenarios simulated for the Kilifi setting

We simulate all combinations of the different parameter values as a grid search (Table 3) and calculate the log likelihoods.

### Implementation

Implementation The individual based stochastic simulation model was created in R statistical software (version 3.02). The simulations were run on sciCORE (http://scicore.unibas.ch/) scientific computing core facility at the University of Basel.

### Ethical approval and informed consent

Ethical approval for the study was obtained from Kenya Medical Research Institute (KEMRI) Ethical Review Committee (under SSC No. 2239). Written informed consent was obtained from parents/guardians of the study participants. The study methods were carried out in accordance with the approved guidelines.

## Results

### Method evaluation

**Figure 2:**
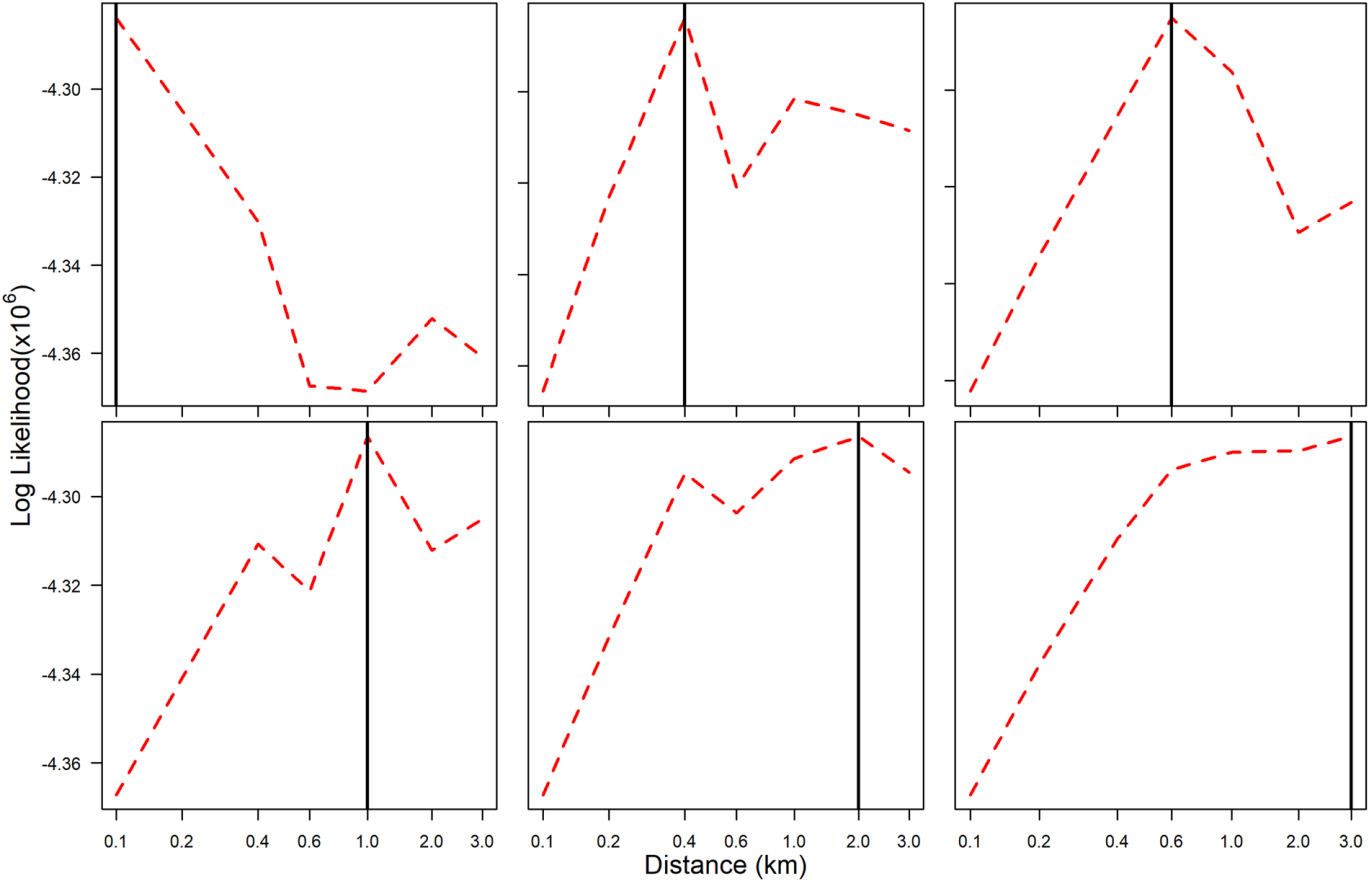
Ability of the method to recover known parameter values from simulated data. Black solid line: indicates the true parameter value of *σ*. Red dashed line: the log likelihood. The log likelihood is a measure of the support from the data for a parameter value. The estimated parameter value is the one which coincides with the maximum log likelihood. In this figure, the estimated parameter values are correct since they align with the black lines. The method and simulated data used the base model with the probability of recombination in multiply infected individuals set to 0.5 (details of the base model are given in the Methods section).

The method was able to recover known values from simulated data for the distance between parent and offspring infections and the probability of recombination (Fig. 2). The model could reproduce reasonable estimates for *σ*, but the estimates were less precise if σ was 2.0 km or greater. For longer distances, the size of the area simulated may limit the accuracy.

### Application to data from Kilifi County

#### Reproducing the number of infections

We were able to reproduce the estimated number of infections in the study area and their decline over the study period by altering the input mean number of new infections per current infection (Supplementary Information Fig. 1). The required value of this input varied by scenario, where longer mean distances and adding immunity functions increased the number of new infections required per current infection per time-step.

#### Model variants and parameter values consistent with the observed data

We fit each model variant to the observed data on the numbers of SNPs different for pairs of parasites. The aim was to determine which model variants and parameter estimates were consistent with the observed interaction between the time and geographic distance.

**Figure 3:**
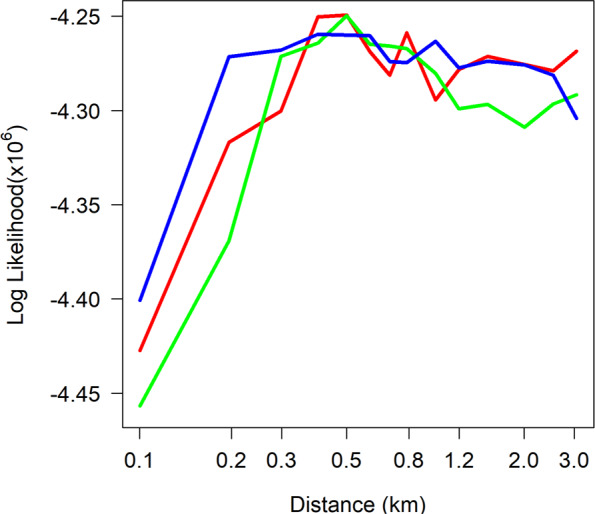
Patterns of the log likelihood for different values of recombination for the base model. The x-axis shows the value of *σ*, the parameter for the distance between parent and offspring infections. The log-likelihood is a measure of support from the data for the parameter values. Red solid line: the base model with probability of recombination in multiply infected individuals set to 0.5; Green line: 0.0; Blue line: 1.0.

Different values for the probability of recombination in multiply infected individuals resulted in similar values for the best-fitting mean distance at the maximum log likelihood (Fig. 3). The probability of recombination had an impact on the log likelihood only for very short mean distances of parasite movement, where it increased variation in the genotypes and increased the log likelihood. Hence, we adopted one value (0.5) for all further analyses shown. The model version allowing four sibling genotypes per recombinant infection produced similar results to those of the base model (Supplementary Information Fig. 2).

The estimates of the mean distance between parent and offspring infections vary slightly by model variant, although the confidence intervals are wide (Table 4).

**Table 4:** Estimated mean distance between parent and offspring infections for each model variant.

Model Variant	Mean distance in km* (95% CI)
Base model	**0.40** (**0.24–1.20)**
Model variant (ii)	**0.32** (**0.24–1.20)**
Model variant (iii)	**0.80** (**0.40–1.99)**
Model variant (iv)	**0.24** (**0.24–1.20)**
Model variant (v)	**0.32** (**0.24–1.20)**

*The mean of a half-normal distribution is given by $\sigma \sqrt{(2/\pi )}$. In all cases, the probability of a short distance is highest and 58% of parent-offspring distances are shorter than the mean value (Fig. 1). Model variant (ii): the base model with imported infections; Model variant (iii): the base model with a limit on the number of current infections per person; Model variant (iv): the base model with immunity to recently seen similar genotypes; Model variant (v): the base model with heterogeneity in transmission at the household level.

**Figure 4:**
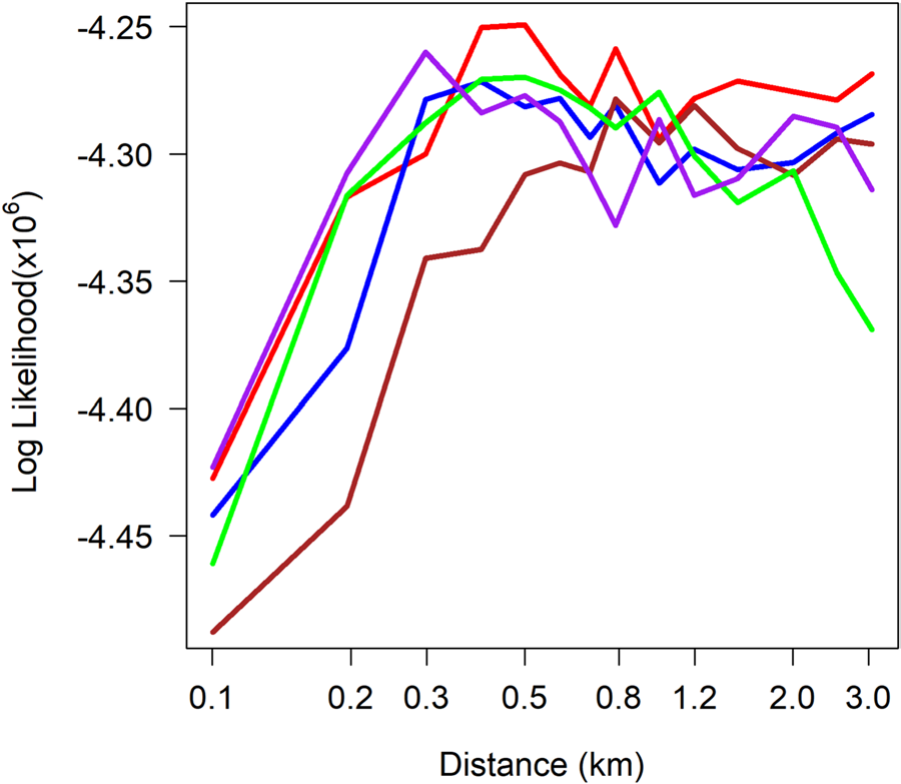
Patterns of the log likelihood by *σ*, the parameter for the distance for the different model variants. Red line: the base model; Blue line: the base model with imported infections (model variant *ii*); Brown line: the base model with a limit on the number of current infections per person (model variant *iii*); Purple line: the base model with immunity to recently seen genotypes (model variant *iv*); Green line: the base model with heterogeneity in transmission at the household level (model variant *v*). Model variant (vi) (combinations of the above variants) are not shown since they produced similar results.

The maximum log likelihood values, a measure of the goodness-of-fit, were similar for the model variants. The differences in the log likelihoods between model variants were greatest for the short mean distances, where the likelihoods tended to increase when the model variants were likely to increase the variation between the genotypes (Fig. 4). While it was not possible to rule out any model variant, the base model alone was sufficient to reproduce the observed patterns. This suggests that stochastic drift is sufficient to account for the observed interaction.

**Figure 5:**
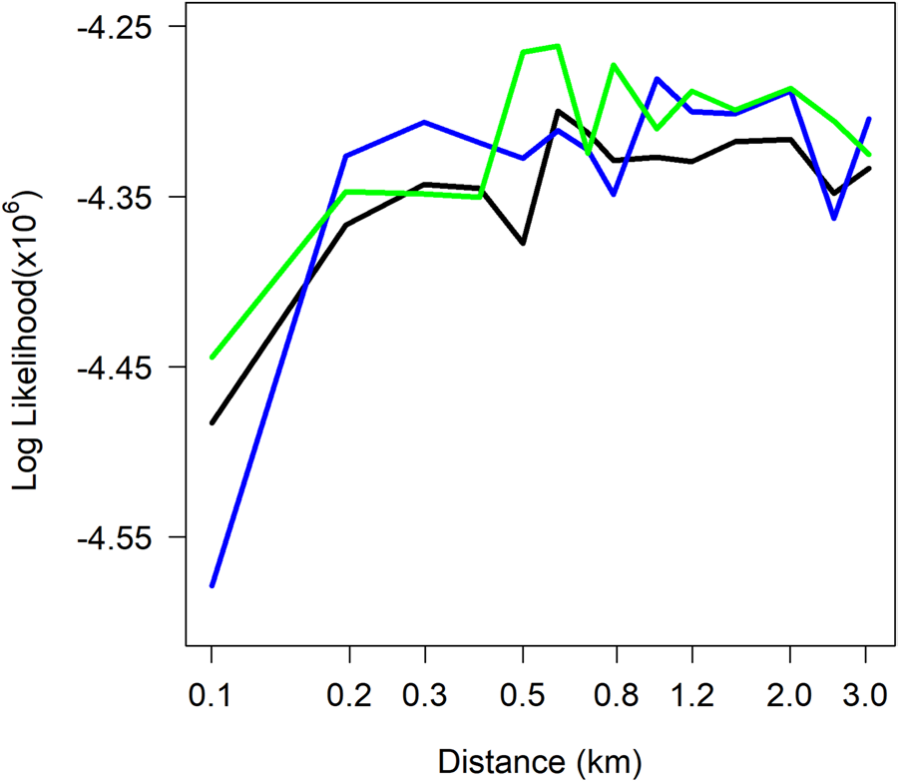
Patterns of the log likelihood by *σ*, the parameter for the distance for data simulated from mixture distributions. Data was simulated assuming a 50:50 mixture distribution for short and longer distances of movement. Black line: 0.05 & 3.0 km; Blue line: 0.1 & 2.5 km; Green line: 0.4 & 4.0 km.

We explored the possibility that the distribution of distances comprises a mixture of short and longer mean distances between parent and offspring infections. This may arise due to a mix of human and vector movement, a mix of local and imported infections, because the normal kernel implicitly assumes the same length of time between the mosquito bites infecting the mosquito and the human and this is likely to vary, or due to the influence of wind or interventions on mosquito dispersal^18^. We simulated data using the base model, but assuming a mixture distribution for the mean distance with half of the distances from a distribution with a short and half from a distribution with a longer mean distance. Applying the method to the simulated data produced a log likelihood profile which was also consistent with the Kilifi data indicating that it is a potential explanation (Fig. 5), although the exact values for the mixture proportions and the two means are unknown and would be difficult to estimate from this data.

#### Reproducing time-distance patterns in observed data

**Figure 6:**
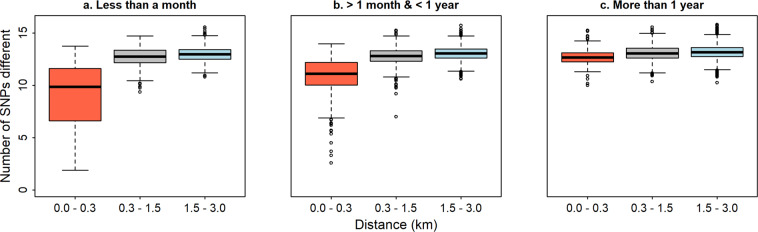
Predicted effect of time and distance on the number of SNPs different between pairs of infections. These predictions are from the base model with the best-fitting value for mean distance (0.4 km).

For each model variant, the observed interaction shown by Omedo and colleagues^3^ could be reproduced for at least some parameter values. The number of SNPs different between pairs of malaria infections increased with geographic distance for short time periods, but this trend was less apparent for longer time periods. The predictions for the base model are shown (Fig. 6), and these patterns were similar for all model variants including the variants of model combinations (ii)–(iv) with imported infections, functions of acquired immunity and heterogeneous transmission at the household level.

**Figure 7:**
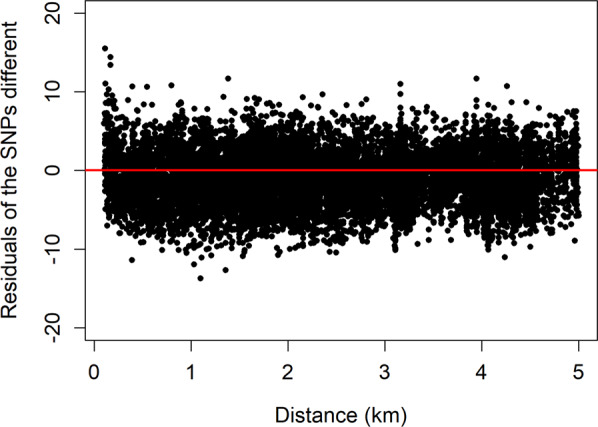
Plot of residuals by geographic distance. The base model was used with the best fitting value for mean geographic distance (0.4 km). We found no evidence of differences in the estimated parameter values between the different 6 km by 6 km squares, or over the course of the study period.

The residual plot showed that the model fitted well with no systematic patterns in the residuals (Fig. 7).

#### Sensitivity analysis of the impact of uncertain input values

**Figure 8:**
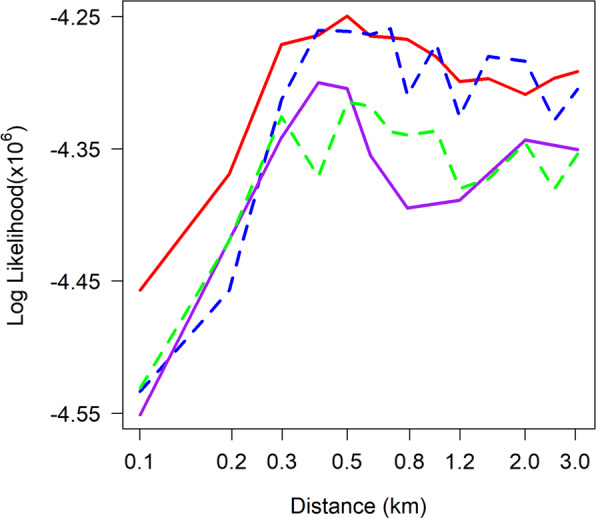
Patterns of the log likelihood by *σ*, the parameter for distance for different values of the input parameters. Red solid line: reference scenario for sensitivity analysis (base model with constant transmission, 13,000 initial infections with only the warm-up and model run-in period, uniform distribution of households), Blue dashed line: reference with a lower number of initial infections (1000 initial infections with an additional run-in period), Green dashed line: reference scenario with half the warm-up period (500 time-steps), Purple solid line: reference with 50% clustering of households in the study area.

We explored the impact of varying the inputs in the base model on the parameter estimates: the number of initial infections, the warm-up period of the simulation, and the degree of clustering of the homesteads. The log likelihood values, measuring how well the model fits, were slightly lower overall for the clustered homesteads and with a shorter warm-up period. A smaller number of initial infections lead to slightly lower log likelihood values for short mean distances, due to the decrease in variation in the genotypes. However none of these factors substantially influenced the estimated distances between parent and offspring infections (Fig. 8). The effect of assuming that the observed genotypes are a mix of alleles from multiple infections within individuals was to increase uncertainty in the parameter estimates (not shown).

## Discussion

We have developed an individual-based stochastic simulation model of malaria infections to determine processes and parameter values which are consistent with the observed patterns of genetic differences in Kilifi County, Kenya. The method was able to recover known parameter values reasonably well from simulated data and could reproduce the number of infections in the study area during the study period.

The observed spatial and temporal patterns of genetic differences could be reproduced for some parameter values for all of the model variants. The log likelihood for the best-fitting parameter values was similar for most of the variants. While we cannot rule out effects of imported infections, genotype-specific acquired immunity or a limit on the number of current infections per person, they were not necessary to account for the observed patterns in the data. This suggests that stochastic drift is a sufficient explanation.

Studies have found that the genotypes of people from the same house are more similar than those from further apart in a village^44–46^ and that index and secondary cases within a radius of 140 m in a study of reactive case detection (RCD) study in Zambia were more genetically similar than cases from the radii of other index cases^17^. One might conclude, based on these and the data of Omedo *et al*., that short distances between parent and offspring infections are more likely. The range of possible values for the mean distance between parent and offspring infections in our study ranged between 0.24 km (0.24 km–1.20 km) and 0.80 km (0.40 km–1.99 km) for the different model variants. These results are consistent with these previous findings, since with the distribution used, 58% of the distances are less than the mean.

Our results also indicated that mixtures of short and longer mean distances were consistent with the observed data, which could potentially represent vector and human movement. It would be challenging to determine the exact mixture of distributions given that the pattern of the log likelihoods is similar for different combinations of values. The mixture of distributions we used to investigate whether this would be consistent with the data was composed of two normal kernels. If one kernel should represent human movement, then another distribution may be more appropriate since it is likely to be incorrect if people only tend to stay elsewhere overnight for distances too far to get home or if human movement tends to follow major roads rather random movements in all directions.

Findings from this study have implications for the assessment and planning of malaria interventions which are affected by parasite movement such as vector control, for setting radii for targeted interventions, for designing trials to evaluate interventions and for informing estimates of the spread of drug resistance^47,48^. While targeting interventions to areas of relatively higher risk on fine scales has been considered^48–50^, our findings highlight the need to consider the movement of infections between untargeted and targeted zones. A modelling study investigating the contribution of RCD towards malaria elimination in Zambia emphasised the need to limit importation of infections from connected high transmission areas, since they could reduce the overall benefits^51^.

There are some limitations to our method. Some of the parameter inputs in the study area were simplified, we did not consider differences according to age in the population, nor did we assume any population turn-over. Our rationale was that this simplifies the model and that although general acquired immunity affects the parasite densities, it does not affect the spatial distribution of genotypes as long as the age-groups are geographically mixed in the population. In one model variant, we account for genotype-specific immunity but this is limited to recent exposure.

During the study period, the area experienced moderate declining to low transmission intensity and the proportion of infections sampled out of the total number in the area was small: we estimate using the reported prevalence^37,38^ and the relationship between prevalence and MOI^31^ that this proportion is less than 1%. In addition, the number of SNPs sampled was not extensive. Therefore measures of identity by descent (IBD)^52–54^ or network models of transmission^13,55^ could not be used.

The sampling may have affected the patterns seen, but this is not known. The samples came from hospital admissions in children aged three months to 13 years and community surveys in individuals aged three weeks to 85 years. We assume that the genotypes in those sampled are no different from those in individuals not sampled. The community surveys were carried out in specific locations but were not otherwise restricted. The hospital sampling may have affected the locations and socio-economic status (SES) of the individuals included. Individuals with higher SES may be over-represented because they may be more likely to present for treatment or under-represented because they may be less likely to have co-morbidity which can increase the risk of severe illness. The distances travelled by the mosquito could potentially be greater in areas of higher SES due to greater protection by LLIN or housing.

This is the first study that we know of which has attempted to estimate parameter values or test hypotheses from malaria genotyping data with a low coverage of infections in a setting with moderate transmission. Few methods are available and although our method is a blunt tool, we have gleaned some insights. Our findings raise questions about whether there is a mixture of more than one distribution for the distances between parent and offspring infections, and a potential influence of immunity on the spatial and temporal distribution patterns of genotypes. It may be possible to gain more definitive results by incorporating additional data sources to isolate mosquito dispersal or human mobility patterns. To fully utilise the information from genotyping tools, purpose-designed datasets have been recommended^5^, ideally with a greater number of SNPs and greater coverage of infections.

## Conclusion

The observed interaction between time and space in the patterns of genetic differences between pairs of *Plasmodium falciparum* infections in Kilifi can be reproduced by an individual-based simulation model. The model did not need any assumptions about genotype-specific acquired immunity or a limit on the number of current infections per person in order to reproduce the patterns, suggesting that stochastic drift is sufficient to account for the interaction.

The estimate of the mean distance between parent and offspring infections was 0.4 km (95% CI 0.24–1.20) for the base model. The pattern of results was also consistent with a mixture of distributions with short and longer mean distances.

Estimates of the spread of infections have implications for the design and evaluation of malaria control and elimination interventions. Simulation models fitted to genotyping data can be used as an analytic tool to glean insights in settings with moderate transmission and a low coverage of infections where methods are limited.

## Supplementary information

Supplementary Information

## Data Availability

The code and data analysed during this study are available at https://github.com/josemalinga/Patterns-of-genetic-differences-in-Kilifi.
