# Author Correction: Investigating the drivers of the spatio-temporal patterns of genetic differences between *Plasmodium falciparum* malaria infections in Kilifi County, Kenya

**DOI:** 10.1038/s41598-020-78848-4

**Published:** 2020-12-23

**Authors:** Josephine Malinga, Polycarp Mogeni, Irene Omedo, Kirk Rockett, Christina Hubbart, Anne Jeffreys, Thomas N. Williams, Dominic Kwiatkowski, Philip Bejon, Amanda Ross

**Affiliations:** 1 Swiss Tropical and Public Health Institute, Basel, Switzerland; 2 University of Basel, Basel, Switzerland; 3 Kenya Medical Research Institute-Wellcome Trust Research Programme, Kilifi, Kenya; 4 Wellcome Trust Centre for Human Genetics, University of Oxford, Oxford, UK; 5 Department of Medicine, South Kensington Campus, Imperial College London, London, UK; 6 Wellcome Trust Sanger Institute, Wellcome Genome Campus, Hinxton, Cambridge, UK; 7 Centre for Tropical Medicine & Global Health, Nuffield Department of Clinical Medicine, University of Oxford, Oxford, UK

Correction to: *Scientific Reports* 10.1038/s41598-019-54348-y, published online 13 December 2019

The original version of this Article contained a typographical error in the spelling of the author Thomas N Williams, which was incorrectly given as Tom Williams.

Consequently, the Author Contributions section now reads:

J.M., P.M., P.B., A.R. conceived and designed the study. J.M., P.M., P.B., A.R. developed the simulation model and analysed the data. I.O., K.R., C.H., A.J., D.K., T.N.W. were involved in sample and/or data collection or genotyping the SNPs. J.M., P.B., A.R., P.M. wrote the first draft of the manuscript. All authors have read and approved the final manuscript.

In addition, the original version of this Article contained a repeated error, where the mean distance was calculated incorrectly. The mean of a half-normal distribution should not be $\sigma$, but $\sqrt {\frac{2}{\pi } } \sigma .$ Therefore, the mean distance between parent and offspring infections should be multiplied by $\sqrt {\frac{2}{\pi } } = 0.798$. These changes do not affect the main conclusions of the original Article.

As a result, in the Abstract,

“The mean geographic distance between parent and offspring malaria infections for the base model was 0.5 km (95% CI 0.3–1.5), for a distribution with 68% of distances shorter than the mean.”

now reads:

“The mean geographic distance between parent and offspring malaria infections for the base model was 0.4 km (95% CI 0.24, 1.20), for a distribution with 58% of distances shorter than the mean.”

In the Methods,

“Therefore, the mean distance between parent and offspring infections is given by *σ*, and 68% of distances are shorter than this value (Fig. 1).”

now reads:

“The mean distance between parent and offspring infections is given by $\sigma \sqrt {2/\pi }$, and 58% of distances are shorter than this value (Fig. 1).”

“We simulated data with known values for the mean distance between parent and offspring infections, *σ*, and probability of recombination, *p*_*r*_, and then applied the method to the simulated data to see how well the known values could be recovered.”

now reads:

“We simulated data with known values for $\sigma$, the parameter for the distance between parent and offspring infections, and probability of recombination, *p*_*r*_, and then applied the method to the simulated data to see how well the known values could be recovered.”

In Table 1,

“mean distance between parent and offspring infection”.

now reads:

“parameter for distance between parent and offspring infection, (mean = $\sigma \sqrt {2/\pi }$).

In Figure 1, the dotted lines marking the mean of distribution were incorrect. As a result the Figure legend,

“Examples of the normal distribution probability density function for positive values of the distance between parent and offspring infections. The dotted lines mark the mean of the distribution; Red line = 0.5 km, Blue line = 1.5 km.”

now reads:

“Examples of the half-normal distribution probability density function for positive values of the distance between parent and offspring infections. The dotted lines mark the mean of the distribution; Red line = 0.4 km, Blue line = 1.2 km.”

In Table 3,

“mean distance between parent and offspring infections (in kilometers)”.

now reads:

“parameter for the distance between parent and offspring infections (in kilometers)**”.

As a result, in the Table legend,

“Simulated scenarios. *The base model scenario is indicated by bold font.”

now reads:

“Simulated scenarios. *The base model scenario is indicated by bold font. ** The mean of a half-normal distribution is given by $\sigma \sqrt {2/\pi } .$ ”

In the Results,

“The method was able to recover known values from simulated data for the mean distance between parent and offspring infections and the probability of recombination (Fig. 2). The model could reproduce reasonable estimates for the mean distance, but the estimates were less precise if the mean distance was 2.0 km or greater. For longer mean distances, the size of the area simulated may limit the accuracy.”

now reads:

“The method was able to recover known values from simulated data for the distance between parent and offspring infections and the probability of recombination (Fig. 2). The model could reproduce reasonable estimates for $\sigma$, but the estimates were less precise if $\sigma$ was 2.0 km or greater. For longer distances, the size of the area simulated may limit the accuracy.”

“The estimates of the mean distance between parent and offspring infections vary slightly by model variant, although the confidence intervals are wide (Table 4).

Model variant (ii): the base model with imported infections; Model variant (iii): the base model with a limit on the number of current infections per person; Model variant (iv): the base model with immunity to recently seen similar genotypes; Model variant (v): the base model with heterogeneity in transmission at the household level.

The maximum log likelihood values, a measure of the goodness-of-fit, were similar for the model variants.”

now reads:

“The estimates of the mean distance between parent and offspring infections vary slightly by model variant, although the confidence intervals are wide (Table 4).

The maximum log likelihood values, a measure of the goodness-of-fit, were similar for the model variants.”

“However none of these factors substantially influenced the estimated mean distance between parent and offspring infections (Fig. 8).”

now reads:

“However none of these factors substantially influenced the estimated distances between parent and offspring infections (Fig. 8).”

In the legend of Figure 2,

“Black solid line: indicates the true parameter value.”

now reads:

“Black solid line: indicates the true parameter value of $\sigma$.”

In the legend of Figure 3,

“The x-axis shows the value of the mean distance between parent and offspring infections.”

now reads:

“The x-axis shows the value of $\sigma$, the parameter for the distance between parent and offspring infections.”

In Table 4, the values “0.5 (0.3–1.5)”, “0.4 (0.3–1.5)”, “1.0 (0.5–2.5)”, “0.3 (0.3–1.5)”, and “0.4 (0.3–1.5)”.

now read:

“0.40 (0.24–1.20)”, “0.32 (0.24–1.20)”, “0.80 (0.40–1.99)”, “0.24 (0.24–1.20)”, and “0.32 (0.24–1.20)”.

As a result, in the Table legend,

“*The mean distance refers to the standard deviation of the positive half of a normal distribution centered around zero. In all cases, the probability of a short distance is highest and 68% of parent–offspring distances are shorter than the mean value (Fig. 1).”

now reads:

“*The mean of a half-normal distribution is given by $\sigma \sqrt {2/\pi }$) . In all cases, the probability of a short distance is highest and 58% of parent–offspring distances are shorter than the mean value (Fig. 1). Model variant (ii): the base model with imported infections; Model variant (iii): the base model with a limit on the number of current infections per person; Model variant (iv): the base model with immunity to recently seen similar genotypes; Model variant (v): the base model with heterogeneity in transmission at the household level.”

In the Discussion,

“The range of possible values for the mean distance between parent and offspring infections in our study ranged between 0.3 km (0.3 km–1.5 km) and 1.0 km (0.5 km–2.5 km) for the different model variants. These results are consistent with these previous findings, since with the distribution used, 68% of the distances are less than the mean and 38% are within half of the mean distance.”

now reads:

“The range of possible values for the mean distance between parent and offspring infections in our study ranged between 0.24 km (0.24 km–1.20 km) and 0.80 km (0.40 km–1.99 km) for the different model variants. These results are consistent with these previous findings, since with the distribution used, 58% of the distances are less than the mean.”

In the legend of Figure 4,

“Patterns of the log likelihood by mean distance for the different model variants.”

now reads:

“Patterns of the log likelihood by $\sigma ,$ the parameter for the distance for the different model variants.”

In the legend of Figure 5,

“Patterns of the log likelihood by mean distance for data simulated from mixture distributions. Data was simulated assuming a 50:50 mixture distribution for short and longer mean distances of movement. Black line: 0.05 & 3.0 km; Blue line: 0.1 & 2.5 km; Green line: 0.4 & 4.0 km.”

now reads:

“Patterns of the log likelihood by ${\upsigma }$, the parameter for the distance for data simulated from mixture distributions. Data was simulated assuming a 50:50 mixture distribution for short and longer distances of movement. Black line: 0.05 & 3.0 km; Blue line: 0.1 & 2.5 km; Green line: 0.4 & 4.0 km.”

In the legend of Figure 6,

“These predictions are from the base model with the best-fitting value for mean distance (0.5 km).”

now reads:

“These predictions are from the base model with the best-fitting value for mean distance (0.4 km).”

In the legend of Figure 7,

“Plot of residuals by geographic distance. The base model was used with the best fitting value for mean geographic distance (0.5 km).”

now reads:

“Plot of residuals by geographic distance. The base model was used with the best fitting value for mean geographic distance (0.4 km).”

In the legend of Figure 8,

“Patterns of the log likelihood by mean distance for different values of the input parameters.”

now reads:

“Patterns of the log likelihood by $\sigma$, the parameter for distance for different values of the input parameters.”

In the Conclusion,

“The estimate of the mean distance between parent and offspring infections was 0.5 km (95% CI 0.3–1.5) for the base model.”

now reads:

“The estimate of the mean distance between parent and offspring infections was 0.4 km (95% CI 0.24–1.20) for the base model.”

Furthermore, in Supplementary Figure 1, the mean distances between parent and offspring infections quoted in the legend should be multiplied by $\sqrt {\frac{2}{\pi } } = 0.798$.

Finally, in Supplementary Figure 2, the X-axis gives the value for $\sigma$ and should be multiplied by $\sqrt {\frac{2}{\pi } } = 0.798$ for the mean.

These errors have now been corrected in the PDF and HTML versions of the Article, and in the accompanying Supplementary Information file.

