# Supplementary material for: Investigating the drivers of the spatio-temporal patterns of genetic differences between Plasmodium falciparum malaria infections in Kilifi County, Kenya
Source: Sci Rep. 2019 Dec 13;9:19018. doi: 10.1038/s41598-019-54348-y (PMC6911066; doi:10.1038/s41598-019-54348-y)
Supplement: Supplementary file 1 — Supplementary Information [file 41598_2019_54348_MOESM1_ESM.docx]

**Supplementary information**

Investigating the drivers of the spatio-temporal patterns of genetic differences between *Plasmodium falciparum* malaria infections in Kilifi County, Kenya

Josephine Malinga, Polycarp Mogeni, Irene Omedo, Kirk Rockett, Christina Hubbart, Anne Jeffreys, Thomas N Williams, Dominic Kwiatkowski, Philip Bejon, Amanda Ross

**Supplementary Figure 1: Estimated and simulated total number of malaria infections in the study area over time
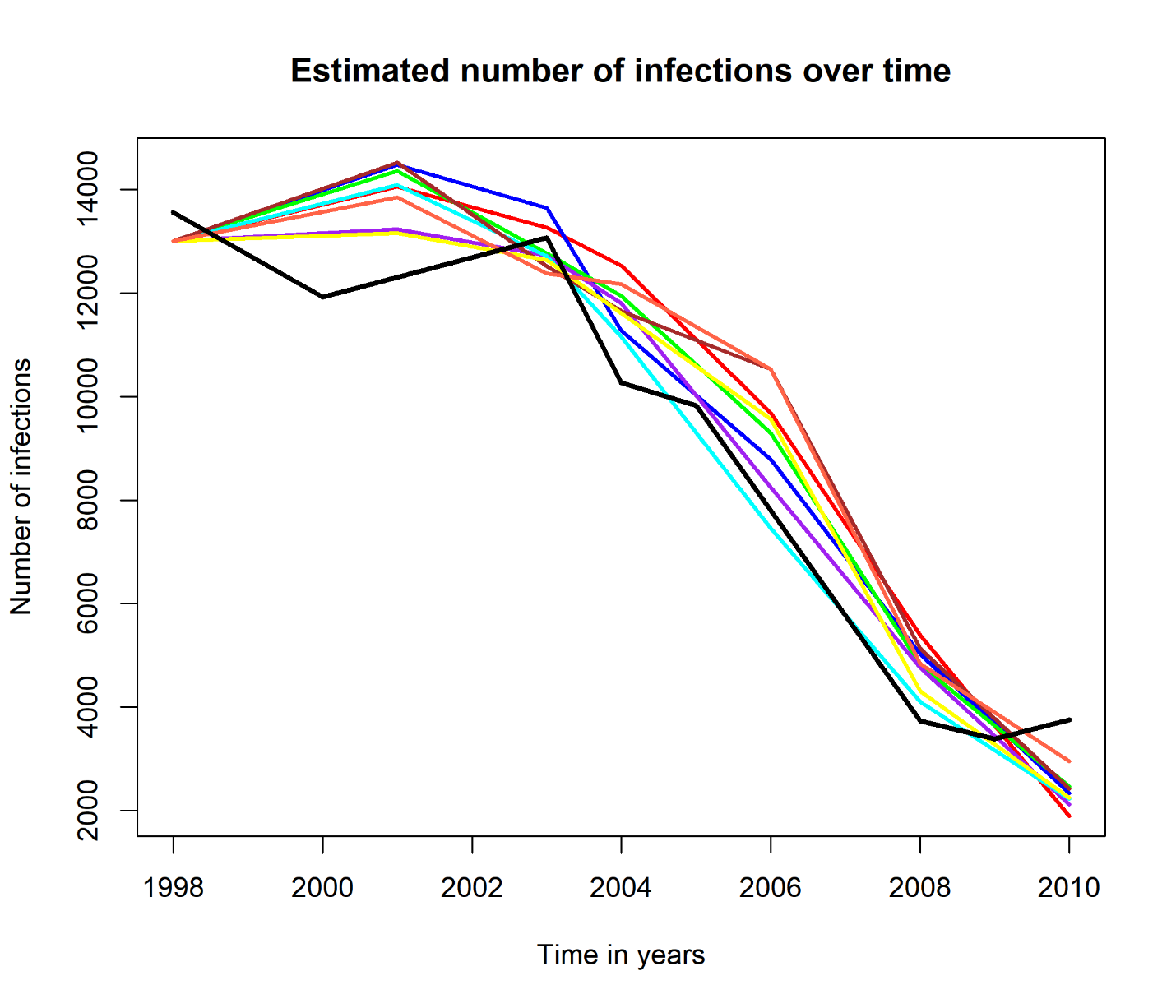
**

Black solid line: the estimated total number of infections in Kilifi over time. The estimated total number of infections is a product of the estimated prevalence in Snow *et al*, population and house density in the Kilifi HDSS, and the multiplicity of infections estimated from Karl *et al.* Red line: the base model with $\sigma,$ the parameter for distance set to 0.1km; Blue line: 0.3km; Green line: 0.4km; Purple line: 0.5km; Cyan line: 0.8km; Brown line: 1.2km; Yellow line: 2.0km Tomato line: 3.0km. (The mean distance is given by $\sigma\sqrt{2/\pi}$, giving 0.24, 0.32, 0.40, 0.64, 0.96, 1.60, 2.39km).

**Supplementary Figure 2: Log likelihood by distance for different assumptions about recombination in multiply infected individuals**

**
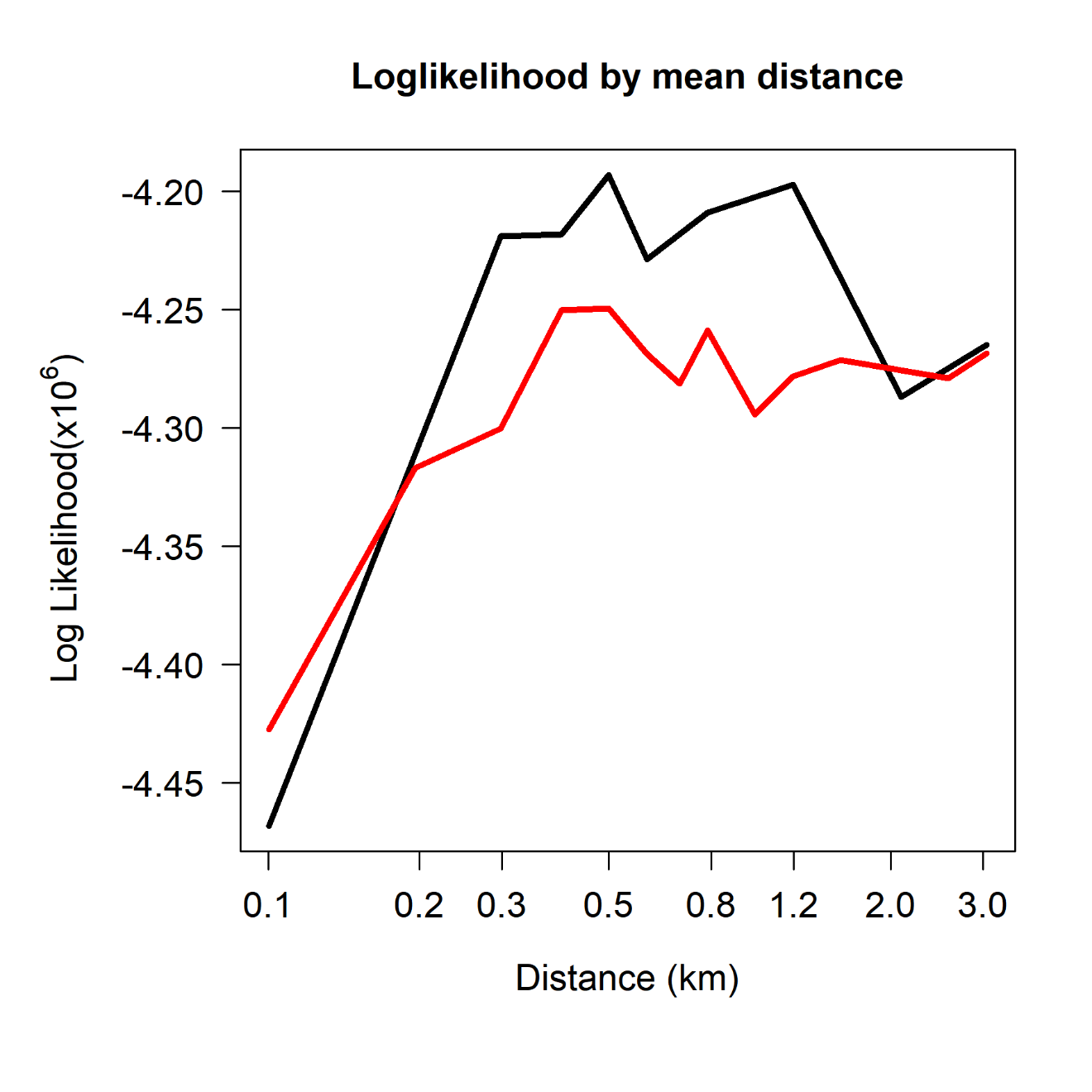
**

The x-axis shows the value of $\sigma,$ the parameter for distance between parent and offspring infections (where the mean distance is given by $\sigma\sqrt{2/\pi}$). The log-likelihood is a measure of support from the data for the parameter values. Red solid line: the base model with probability of recombination in multiply infected individuals set to 0.5; Black line: the base model with probability of recombination in multiply infected individuals set to 0.5 and allowing four sibling genotypes per recombinant infection.
